# Crystal structure and computational study of 2,4-di­chloro-*N*-[(*E*)-(5-nitro­thio­phen-2-yl)methyl­idene]aniline

**DOI:** 10.1107/S2056989016011816

**Published:** 2016-07-22

**Authors:** Yavuz Köysal, Hakan Bülbül, Sümeyye Gümüş, Erbil Ağar, Mustafa Serkan Soylu

**Affiliations:** a Yesilyurt Demir Celik Vocational School, Ondokuz Mayıs University, TR-55139 Samsun, Turkey; b Department of Physics, Faculty of Arts and Sciences, Ondokuz Mayıs University, TR-55139 Samsun, Turkey; c Department of Chemistry, Faculty of Arts and Sciences, Ondokuz Mayıs University, Kurupelit, 55139 Samsun, Turkey; d Department of Physics, Faculty of Arts and Sciences, Giresun University, Giresun, Turkey

**Keywords:** crystal structure, Schiff base, nitro­thio­phene, π–π inter­actions, quantum-chemical calculations

## Abstract

The title compound, C_11_H_6_Cl_2_N_2_O_2_S, is a Schiff base that incorporates an N-bound 2,4-di­chloro­phenyl and a C-bound 5-nitro­thio­phene ring. The mol­ecule is approximately planar, the maximum deviation from the mean plane being 0.233 (4) Å for the C=N N atom. The dihedral angle between the benzene and thio­phene rings is 9.7 (2)°. The C=N double bond has an *E* configuration. The crystal structure features C—H⋯O hydrogen bonds,forming sheets parallel to (10-1), and π–π stacking inter­actions between symmetry-related thio­phene and benzene rings, in which the distance between adjacent ring centroids is 3.707 (4) Å, forming a three-dimensional supramolecular structure. Geometric parameters from quantum-chemical calculations are in good agreement with experimental X-ray diffraction results.

## Abstract 2

The title compound, C_11_H_6_Cl_2_N_2_O_2_S, is a Schiff base that incorporates an N-bound 2,4-di­chloro­phenyl and a C-bound 5-nitro­thio­phene ring. The crystal structure features C—H⋯O hydrogen bonds and π–π stacking inter­actions. Geometric parameters from quantum-chemical calculations are in good agreement with experimental X-ray diffraction results.

## Chemical context   

Schiff bases, which contain C=N double bonds, are well known starting materials for the synthesis of many drugs (Aydoğan *et al.*, 2001 ▸) and often possess very important biological activities, such as anti-inflammatory and analgesic properties (Sondhi *et al.*, 2006 ▸). In addition, nitro­thio­phene and its derivatives also exhibit many biological activities, including anti­bacterial and anti­fungal (Kalluraya *et al.*, 1994 ▸; Kalluraya & Shetty, 1997 ▸) properties. We report the synthesis, structural analysis and theoretical calculations of the title compound, C_11_H_6_Cl_2_N_2_O_2_S (I), which is a new Schiff base that includes a nitro­thio­phene group.
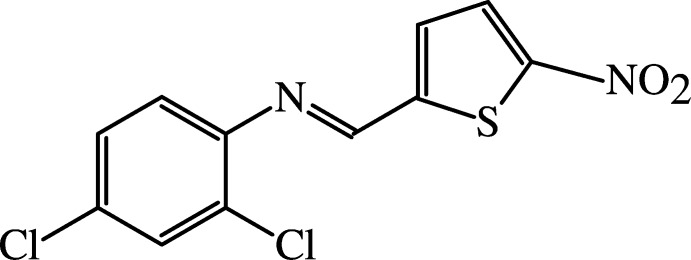


## Structural commentary   

**Figure 1:**
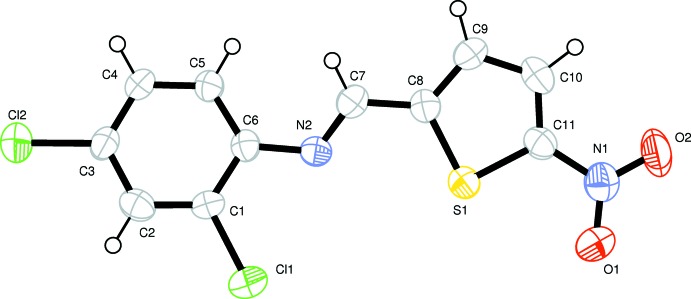
A view of (I), with the atom-numbering scheme and 50% probability displacement ellipsoids.

The title compound (Fig. 1 ▸) is nearly planar, the maximum deviation from the mean plane of 0.233 (4) Å is for atom N2. Schiff bases that are derived from salicyl­aldehyde show thermochromism and photochromism properties that are dependent upon planarity or non-planarity of the mol­ecules (Cohen *et al.*, 1964 ▸; Hadjoudis *et al.*, 1987 ▸). Since the dihedral angle between the benzene and thio­phene rings is 9.7 (2)°, the title compound may exhibit thermochromic features. The slight twist of the mol­ecule is caused by a steric repulsion of atoms H5 and H7. The C7=N2 double-bond distance is 1.267 (6)Å, which is comparable to those of reported structures (Özdemir Tarı & Işık, 2012 ▸; Ceylan *et al.*, 2012 ▸). The C8—C7—N2—C6 torsion angle is 178.5 (5)°.

## Supra­molecular features   

**Figure 2:**
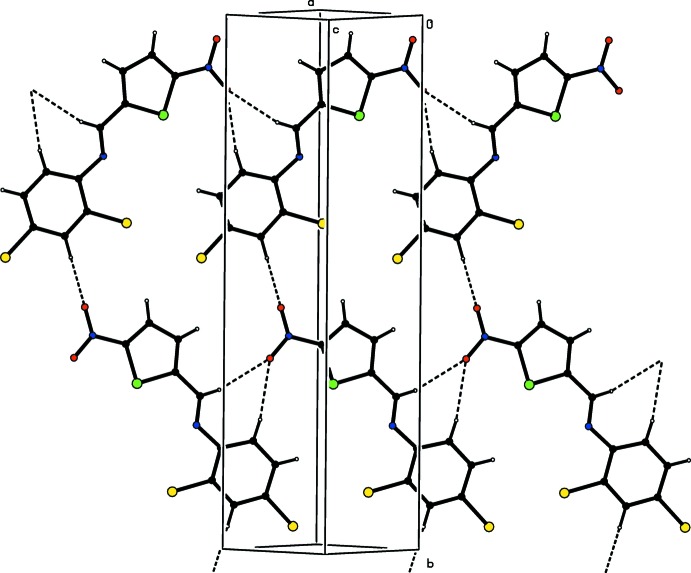
A partial packing view of (I). Dashed lines indicate the C—H⋯O hydrogen-bonding inter­actions

In the crystal structure there are weak C—H⋯O hydrogen bonds (Fig. 2 ▸ and Table 1 ▸) with atom O1 acting as a bifurcated acceptor from both C5 and C7 (*x* − 1, y, *z* − 1), creating an 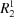
(7) motif, and forming sheets parallel to (10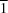). π–π stacking inter­actions are present between the benzene (centroid *Cg*2) and thio­phene (centroid *Cg*1) rings of symmetry-related mol­ecules [*Cg*1⋯*Cg2*(*x*, 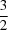 − *y*, 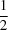 + *z*) = 3.707 (4) Å, forming a three-dimensional supramolecular structure.

## Theoretical Calculations   

Quantum-chemical calculations were performed to compare with the experimental analysis. *Ab initio* Hartree–Fock (HF) and density functional DFT(B3LYP) methods were used with the standard basis set of 6-31+G(d) (Becke, 1993 ▸; Lee *et al.*, 1988 ▸; Schlegel, 1982 ▸; Peng *et al.*, 1996 ▸) using the *Gaussian 03* software package (Frisch *et al.*, 2004 ▸; Dennington *et al.*, 2007 ▸) to obtain the optimized mol­ecular structure. The computational results are consistent with experimental crystallographic data. The C7=N2 bond length was calculated to be 1.25 and 1.28 Å using HF and DFT(B3LYP) methods, respectively. The torsion angle C8—C7—N2—C6 was calculated to be −177.98 and −176.09° by HF and DFT(B3LYP) methods, respectively.

## Synthesis and crystallization   

The compound 2,4-di­chloro-*N*-[(*E*)-(5-nitro­thio­phen-2-yl)methyl­idene]aniline was prepared by refluxing a mixture of a solution containing 5-nitro-2-thio­phene­carboxaldehyde (0.0180 g, 0.114 mmol) in 20 ml ethanol and a solution containing 2,4-di­chloro­aniline (0.0185 g, 0.114 mmol) in 20 ml ethanol. The reaction mixture was stirred for 1h under reflux. Crystals suitable for X-ray analysis were obtained from a solution in ethanol by slow evaporation (yield 65%; m.p 443–445 K).

IR (KBr/cm^−1^): 3102.59 (C—H), 1602.71 (C=N), 1503.00 (NO_2_), 1231.00 (C—N, methyl­ene), 1192.05 (C—N, thio­phene), 1039.60 (C—H, thio­phene), 1124.10 (C—H, methyl­ene), 957.96 (C—H, methyl­ene), 787.73 (C—H, 957.96)

## Refinement   

Crystal data, data collection and structure refinement details are summarized in Table 2 ▸. All H atoms were positioned geometrically with C—H = 0.93 Å and refined with using a riding model with *U*_iso_(H) = 1.2*U*_eq_(C).

## Supplementary Material

Crystal structure: contains datablock(s) I. DOI: 10.1107/S2056989016011816/pk2586sup1.cif

Structure factors: contains datablock(s) I. DOI: 10.1107/S2056989016011816/pk2586Isup2.hkl

Supporting information file. DOI: 10.1107/S2056989016011816/pk2586Isup3.cml

CCDC reference: 1494850

Additional supporting information: crystallographic information; 3D view; checkCIF report

## Figures and Tables

**Table 1:** Hydrogen-bond geometry (Å, °)

*D*—H⋯*A*	*D*—H	H⋯*A*	*D*⋯*A*	*D*—H⋯*A*
C5—H5⋯O1^i^	0.93	2.59	3.508 (8)	171
C7—H7⋯O1^i^	0.93	2.55	3.300 (8)	138
C2—H2⋯O2^ii^	0.93	2.56	3.360 (7)	144

Symmetry codes: (i) 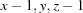; (ii) 
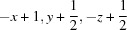
.

**Table 2:** Experimental details

Crystal data	
Chemical formula	C_11_H_6_Cl_2_N_2_O_2_S
*M*_r_	301.14
Crystal system, space group	Monoclinic, *P*2_1_/*c*
Temperature (K)	293
*a*, *b*, *c* (Å)	7.5731 (9), 22.1795 (16), 8.3093 (16)
β (°)	117.967 (10)
*V* (Å^3^)	1232.7 (3)
*Z*	4
Radiation type	Mo *K*α
μ (mm^−1^)	0.69
Crystal size (mm)	0.18 × 0.15 × 0.10

Data collection	
Diffractometer	Agilent SuperNova (Single source at offset) Eos
Absorption correction	Multi-scan (*CrysAlis PRO*; Agilent, 2011)
*T*_min_, *T*_max_	0.712, 1.000
No. of measured, independent and observed [*I* > 2σ(*I*)] reflections	3059, 2201, 1119
*R*_int_	0.033
(sin θ/λ)_max_ (Å^−1^)	0.617

Refinement	
*R*[*F* ^2^ > 2σ(*F* ^2^)], *wR*(*F* ^2^), *S*	0.063, 0.138, 1.09
No. of reflections	2201
No. of parameters	163
H-atom treatment	H-atom parameters constrained
Δρ_max_, Δρ_min_ (e Å^−3^)	0.38, −0.32

Computer programs: *CrysAlis PRO* (Oxford Diffraction, 2007 ▸), *SHELXS97* (Sheldrick, 2008 ▸), *SHELXL2014* (Sheldrick, 2015 ▸) and *ORTEP-3 for Windows* and *WinGX* (Farrugia, 2012 ▸).

## supplementary crystallographic information

### Crystal data

**Table:**

C_11_H_6_Cl_2_N_2_O_2_S	*F*(000) = 608
*M**_r_* = 301.14	*D*_x_ = 1.623 Mg m^−^^3^
Monoclinic, *P*2_1_/*c*	Mo *K*α radiation, λ = 0.71073 Å
*a* = 7.5731 (9) Å	Cell parameters from 612 reflections
*b* = 22.1795 (16) Å	θ = 3.2–28.4°
*c* = 8.3093 (16) Å	µ = 0.69 mm^−^^1^
β = 117.967 (10)°	*T* = 293 K
*V* = 1232.7 (3) Å^3^	Block, red
*Z* = 4	0.18 × 0.15 × 0.10 mm

### Data collection

**Table:**

Agilent SuperNova (Single source at offset) Eos diffractometer	2201 independent reflections
Radiation source: SuperNova (Mo) X-ray Source	1119 reflections with *I* > 2σ(*I*)
Detector resolution: 16.0454 pixels mm^-1^	*R*_int_ = 0.033
ω scans	θ_max_ = 26.0°, θ_min_ = 3.2°
Absorption correction: multi-scan (CrysAlis PRO; Agilent, 2011)	*h* = −7→9
*T*_min_ = 0.712, *T*_max_ = 1.000	*k* = −27→12
3059 measured reflections	*l* = −10→5

### Refinement

**Table:**

Refinement on *F*^2^	0 restraints
Least-squares matrix: full	Hydrogen site location: inferred from neighbouring sites
*R*[*F*^2^ > 2σ(*F*^2^)] = 0.063	H-atom parameters constrained
*wR*(*F*^2^) = 0.138	*w* = 1/[σ^2^(*F*_o_^2^) + (0.019*P*)^2^] where *P* = (*F*_o_^2^ + 2*F*_c_^2^)/3
*S* = 1.09	(Δ/σ)_max_ < 0.001
2201 reflections	Δρ_max_ = 0.38 e Å^−^^3^
163 parameters	Δρ_min_ = −0.32 e Å^−^^3^

### Special details

**Table:**

Geometry. All esds (except the esd in the dihedral angle between two l.s. planes) are estimated using the full covariance matrix. The cell esds are taken into account individually in the estimation of esds in distances, angles and torsion angles; correlations between esds in cell parameters are only used when they are defined by crystal symmetry. An approximate (isotropic) treatment of cell esds is used for estimating esds involving l.s. planes.

### Fractional atomic coordinates and isotropic or equivalent isotropic displacement parameters (Å^2^)

**Table:**

	*x*	*y*	*z*	*U*_iso_*/*U*_eq_	
S1	0.4933 (2)	0.69183 (7)	0.38617 (19)	0.0450 (4)	
Cl1	0.3105 (3)	0.89412 (7)	0.2025 (2)	0.0612 (5)	
Cl2	−0.2711 (3)	0.95859 (7)	−0.4521 (2)	0.0720 (6)	
C6	0.0675 (8)	0.8117 (2)	−0.0461 (7)	0.0369 (14)	
C7	0.1939 (9)	0.7153 (3)	0.0411 (7)	0.0473 (16)	
H7	0.1199	0.7042	−0.0806	0.057*	
C5	−0.0926 (8)	0.7988 (3)	−0.2169 (7)	0.0443 (15)	
H5	−0.1307	0.7588	−0.2472	0.053*	
C2	0.0113 (9)	0.9185 (3)	−0.1289 (7)	0.0468 (16)	
H2	0.0445	0.9588	−0.0990	0.056*	
C4	−0.1955 (9)	0.8429 (3)	−0.3414 (7)	0.0446 (15)	
H4	−0.3005	0.8328	−0.4545	0.053*	
O1	0.8046 (7)	0.6438 (2)	0.7224 (6)	0.0768 (15)	
N2	0.1847 (7)	0.7692 (2)	0.0870 (6)	0.0429 (12)	
C10	0.4560 (10)	0.5791 (3)	0.3021 (8)	0.0499 (16)	
H10	0.4750	0.5376	0.3102	0.060*	
N1	0.7237 (8)	0.6023 (2)	0.6127 (7)	0.0538 (14)	
C1	0.1141 (8)	0.8726 (2)	−0.0056 (7)	0.0395 (14)	
O2	0.7734 (7)	0.5493 (2)	0.6444 (6)	0.0761 (15)	
C9	0.3146 (9)	0.6094 (3)	0.1475 (7)	0.0495 (17)	
H9	0.2288	0.5902	0.0393	0.059*	
C11	0.5608 (9)	0.6182 (3)	0.4372 (8)	0.0450 (15)	
C8	0.3165 (9)	0.6706 (3)	0.1730 (7)	0.0445 (15)	
C3	−0.1422 (9)	0.9020 (3)	−0.2976 (7)	0.0436 (15)	

### Atomic displacement parameters (Å^2^)

**Table:**

	*U*^11^	*U*^22^	*U*^33^	*U*^12^	*U*^13^	*U*^23^
S1	0.0440 (10)	0.0394 (9)	0.0480 (9)	0.0004 (8)	0.0184 (8)	0.0007 (7)
Cl1	0.0617 (12)	0.0609 (11)	0.0451 (9)	−0.0060 (9)	0.0118 (9)	−0.0096 (8)
Cl2	0.0749 (15)	0.0527 (10)	0.0616 (11)	0.0061 (10)	0.0096 (11)	0.0135 (9)
C6	0.041 (4)	0.038 (3)	0.036 (3)	0.004 (3)	0.022 (3)	0.004 (3)
C7	0.051 (4)	0.050 (4)	0.037 (3)	0.000 (3)	0.017 (3)	0.002 (3)
C5	0.037 (4)	0.039 (3)	0.046 (3)	0.003 (3)	0.009 (3)	0.002 (3)
C2	0.051 (4)	0.042 (4)	0.053 (4)	−0.009 (3)	0.029 (4)	−0.003 (3)
C4	0.041 (4)	0.047 (4)	0.033 (3)	0.001 (3)	0.007 (3)	0.001 (3)
O1	0.069 (4)	0.067 (3)	0.060 (3)	−0.008 (3)	0.001 (3)	−0.003 (3)
N2	0.042 (3)	0.037 (3)	0.045 (3)	−0.005 (2)	0.016 (3)	−0.001 (2)
C10	0.064 (5)	0.036 (3)	0.060 (4)	0.006 (3)	0.038 (4)	0.003 (3)
N1	0.049 (4)	0.055 (4)	0.061 (4)	0.004 (3)	0.029 (3)	0.007 (3)
C1	0.037 (4)	0.044 (4)	0.036 (3)	−0.006 (3)	0.016 (3)	−0.004 (3)
O2	0.086 (4)	0.053 (3)	0.082 (3)	0.028 (3)	0.033 (3)	0.021 (2)
C9	0.052 (5)	0.053 (4)	0.036 (3)	−0.001 (3)	0.014 (3)	−0.001 (3)
C11	0.049 (4)	0.039 (3)	0.049 (4)	−0.004 (3)	0.025 (4)	0.002 (3)
C8	0.042 (4)	0.042 (3)	0.046 (4)	0.003 (3)	0.018 (3)	0.003 (3)
C3	0.041 (4)	0.049 (4)	0.033 (3)	0.003 (3)	0.011 (3)	0.005 (3)

### Geometric parameters (Å, º)

**Table:**

S1—C11	1.703 (6)	C2—C1	1.394 (7)
S1—C8	1.711 (6)	C2—H2	0.9300
Cl1—C1	1.737 (5)	C4—C3	1.370 (7)
Cl2—C3	1.732 (6)	C4—H4	0.9300
C6—C5	1.397 (7)	O1—N1	1.237 (6)
C6—C1	1.398 (7)	C10—C11	1.344 (7)
C6—N2	1.407 (6)	C10—C9	1.399 (7)
C7—N2	1.267 (6)	C10—H10	0.9300
C7—C8	1.446 (7)	N1—O2	1.226 (6)
C7—H7	0.9300	N1—C11	1.445 (7)
C5—C4	1.371 (7)	C9—C8	1.372 (7)
C5—H5	0.9300	C9—H9	0.9300
C2—C3	1.386 (7)		
			
C11—S1—C8	89.5 (3)	C9—C10—H10	124.6
C5—C6—C1	116.3 (5)	O2—N1—O1	124.1 (6)
C5—C6—N2	126.1 (5)	O2—N1—C11	118.8 (5)
C1—C6—N2	117.6 (5)	O1—N1—C11	117.1 (5)
N2—C7—C8	121.7 (5)	C2—C1—C6	122.5 (5)
N2—C7—H7	119.2	C2—C1—Cl1	117.0 (4)
C8—C7—H7	119.2	C6—C1—Cl1	120.4 (4)
C4—C5—C6	122.5 (6)	C8—C9—C10	112.6 (5)
C4—C5—H5	118.8	C8—C9—H9	123.7
C6—C5—H5	118.8	C10—C9—H9	123.7
C3—C2—C1	117.8 (5)	C10—C11—N1	125.2 (6)
C3—C2—H2	121.1	C10—C11—S1	114.8 (5)
C1—C2—H2	121.1	N1—C11—S1	120.0 (4)
C3—C4—C5	119.3 (5)	C9—C8—C7	127.2 (6)
C3—C4—H4	120.3	C9—C8—S1	112.2 (4)
C5—C4—H4	120.3	C7—C8—S1	120.6 (5)
C7—N2—C6	119.9 (5)	C4—C3—C2	121.6 (5)
C11—C10—C9	110.8 (6)	C4—C3—Cl2	120.2 (4)
C11—C10—H10	124.6	C2—C3—Cl2	118.2 (5)
			
C1—C6—C5—C4	2.0 (8)	O1—N1—C11—C10	−179.9 (6)
N2—C6—C5—C4	−177.9 (5)	O2—N1—C11—S1	−177.9 (5)
C6—C5—C4—C3	−0.7 (9)	O1—N1—C11—S1	2.2 (7)
C8—C7—N2—C6	178.5 (5)	C8—S1—C11—C10	−0.3 (5)
C5—C6—N2—C7	22.1 (9)	C8—S1—C11—N1	177.9 (5)
C1—C6—N2—C7	−157.7 (5)	C10—C9—C8—C7	178.5 (5)
C3—C2—C1—C6	−0.2 (8)	C10—C9—C8—S1	0.6 (7)
C3—C2—C1—Cl1	178.2 (4)	N2—C7—C8—C9	168.4 (6)
C5—C6—C1—C2	−1.5 (8)	N2—C7—C8—S1	−13.8 (8)
N2—C6—C1—C2	178.4 (5)	C11—S1—C8—C9	−0.2 (5)
C5—C6—C1—Cl1	−179.9 (3)	C11—S1—C8—C7	−178.3 (5)
N2—C6—C1—Cl1	0.0 (7)	C5—C4—C3—C2	−1.2 (9)
C11—C10—C9—C8	−0.8 (8)	C5—C4—C3—Cl2	−179.6 (4)
C9—C10—C11—N1	−177.4 (5)	C1—C2—C3—C4	1.6 (8)
C9—C10—C11—S1	0.6 (7)	C1—C2—C3—Cl2	−180.0 (4)
O2—N1—C11—C10	0.0 (9)		

### Hydrogen-bond geometry (Å, º)

**Table:**

*D*—H···*A*	*D*—H	H···*A*	*D*···*A*	*D*—H···*A*
C5—H5···O1^i^	0.93	2.59	3.508 (8)	171
C7—H7···O1^i^	0.93	2.55	3.300 (8)	138
C2—H2···O2^ii^	0.93	2.56	3.360 (7)	144

Symmetry codes: (i) *x*−1, *y*, *z*−1; (ii) −*x*+1, *y*+1/2, −*z*+1/2.
